# Extensive anogenital warts: a multidisciplinary surgical management

**DOI:** 10.11604/pamj.2018.30.227.16448

**Published:** 2018-07-26

**Authors:** Kostas Chondros, Konstantinos Graikos, Athanasios Klambatsas, Nikolaos Dimasis

**Affiliations:** 1 Department of Urology, General Hospital of Rethymnon, Rethymnon, Greece; 2 Department of Urology-Oncology, “Theageneio” Cancer Hospital of Thessaloniki, Thessaloniki, Greece

**Keywords:** Anogenital warts, condylomata acuminata, anus obstruction, HPV infection

## Abstract

Genital warts are a major worldwide healthcare problem of sexually active population. Apart from direct association with cervical cancer, male patients experience HPV-related condylomata in several locations in the anogenital area. Extensive growth, multifocality and difficult accessibility wart sites are demanding and often require multidisciplinary surgical management. In our case, we present a male patient with extensive anogenital warts treated by a team of surgeons in several steps.

## Introduction

Condylomata acuminata constitute papillary exophytic lesions that are often found in the anogenital area (glans, meatus, anus and penis). Less commonly they can be located within the urethra, scrotum, pubic area and crurogenital aspects. Genital warts are closely associated with Human papillomavirus (HPV) infection and they are considered a sexually transmitted disease. Typically, they are characterized by discrete verrucose or papillary growths and occasionally, functional, cosmetic and hygiene problems can arise due to their extensive size [1]. In this case report, we present a male patient with extensive and huge warts in multiple locations around the anogenital area that needed several surgical interventions to achieve a good cosmetic and functional result by a multidisciplinary team.

## Patient and observation

**Figure 1:**
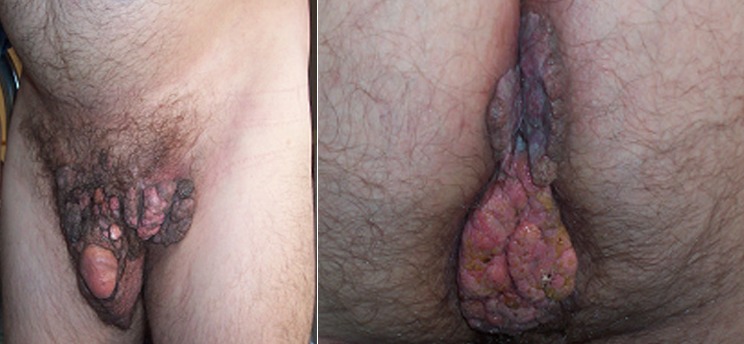
Clinical appearance of extensive warts in the suprapubic area, penis and anal area

**Figure 2:**
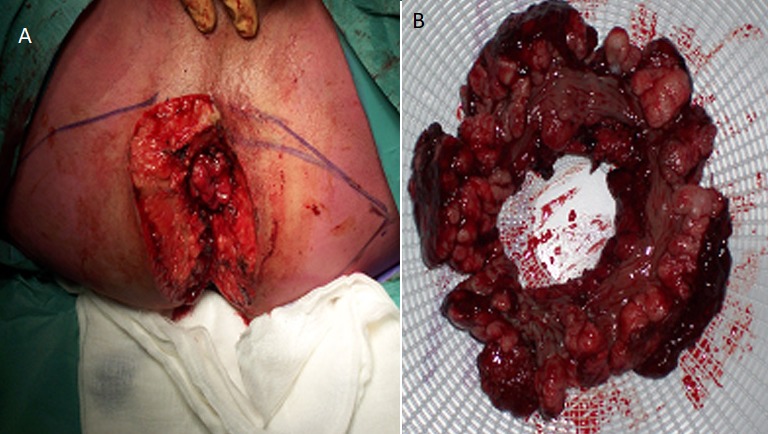
A) operative image of the large surgical dissection of the perianal lesions creating a skin deficit; B) the circumferential surgical specimen

**Figure 3:**
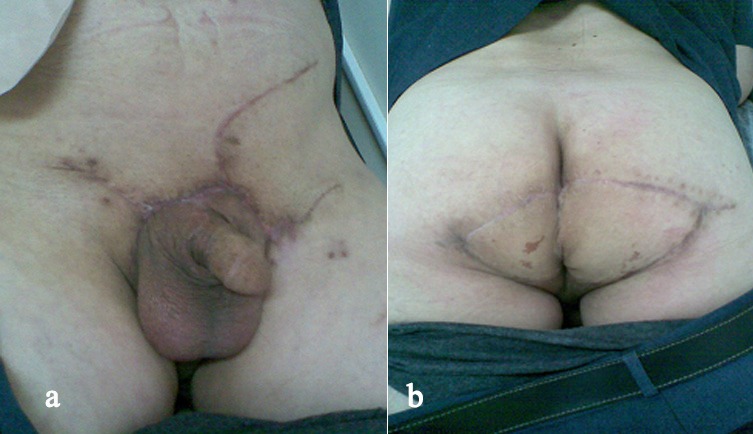
Post-surgery results at 1-year follow-up with minor scars from multiple skin flaps transpositions; (A) Pubic area and penis; (B) peri-anal area

A 61-year-old male presented in our hospital (Department of Urology-Oncology, “Theageneio” Cancer Hospital of Thessaloniki, Greece) with extensive genital warts located in the pubic area, inguinal area, penis and peri-anal region, complaining about defecation difficulty (Figure 1). The patient was aware of the presence of these warts for 15 years but never submitted to any therapy. The patient underwent laboratory tests including biochemical tests and virology assessment for HBC, HCV and HIV. A colonoscopy was planned in order to investigate the bowel obstruction, which was negative for any lesions beyond the anal area and a biopsy was taken to confirm the diagnosis of HPV-related genital warts, pathologically. As a result and due to persistent anal pseudo-obstruction from the extensive anal ring lesions, a surgical transverse colostomy was deemed necessary, by the colorectal surgeon. Subsequently, all anal defects including infected peri-anal skin were removed, while rectal mucosa was preserved and the area was reconstructed using two transposition flaps (Figure 2). Three months later, the patient was submitted to surgical removal of the lesions on the pubic area and penis, by the Urologic Surgeon and with the assistance of a Plastic Surgeon, the large skin deficit was restored using skin flaps. Then, four months later, the patient underwent again a second resection of the peri-anal tissue due to anal stenosis that didn't allow any endoscopic evaluation. The deficit from the circumferential resected scarring tissue was closed anew, using a z-plastic technique for maximum cosmetic results (Figure 3). Three months later the bowel ostomy was restored. Despite their massive size, the lesions were completely healed and the patient didn't have any recurrence at 1 year of follow-up.

## Discussion

HPV infection is one of the most common sexually transmitted decease amongst the young population which represents a 20% of prevalence in males [2] and can reach 90% in cases of homosexual men. In women, it is a well-established causative factor for cervical cancer and in men, it is strongly related, especially HPV 16 and 18 subtypes, with penile, oral and anal cancers up to 90% [3]. Condylomata acuminata (also known as warts) are non-malignant HPV-associated lesions are usually associated with HPV types 6 and 11 but may have a malignant potential. They can be found in several sites apart from the cervix, such as anus, vagina, penis, pubic and perianal area, mouth and pharynx and represent mucocutaneous lesions with slow development (6-10 months) [4]. In most of the cases, the diagnosis is clinical and histopathologic examination is often used to confirm the diagnosis and rule out squamous cell carcinomas. In general, treatment options include topical cream appliance with several substances, cryotherapy, laser therapy or surgical removal of any visible lesion [5]. HPV vaccination programs running the last decade for school-aged girls and boys have managed to reduce anogenital warts by 62%, cervical cancer by 45% and anal cancer by 54% [6]. In certain rare cases, warts may gain giant sizes (Buschke - Loewenstein tumor) and create a treatment challenge. In these cases, surgical treatment is the only option due to extensive size of warts that creates large skin defects [7]. As in our case, multiple and extensive resections where needed to excise completely all the lesions, while skin plastic reconstruction is essential for the best functional and cosmetic result. Anal pseudo-obstruction is a rare complication of massive anal wart growth. In addition, anal stricture development after extensive surgical excision of anal warts is rarely present [8], but in our case, the anal obstruction was initially present and circumferential resection contributed to secondary anal stricture. Skin transpositioned flaps are the best option to manage large skin defects. Finally, close follow-up is mandatory due to a relevant risk of recurrence, which in cases of dissection reaches 6% [9].

## Conclusion

Anogenital HPV-related warts are a very common disease in the general population with high prevalence. Treatment can be sometimes challenging especially in cases with severe size growth and complicate clinical presentation such as multifocality, or anal obstruction that requires a multidisciplinary approach.

## Competing interests

The authors declare no competing interest.
